# Comparison of Clinical, Laboratory, and Ultrasonographic Findings in Dogs With Acutely Presenting Clinical Signs and Either Normal or Increased Serum DGGR Lipase Activity

**DOI:** 10.1111/jvim.70134

**Published:** 2025-06-01

**Authors:** Melanie Sidler, Daniel Brugger, Barbara Riond, Matthias Dennler, Stefan Unterer, Peter H. Kook

**Affiliations:** ^1^ Clinic for Small Animal Internal Medicine, Vetsuisse Faculty University of Zurich Zurich Switzerland; ^2^ Institute for Animal Nutrition and Dietetics, Vetsuisse Faculty University of Zurich Zurich Switzerland; ^3^ Clinical Laboratory, Vetsuisse Faculty University of Zurich Zurich Switzerland; ^4^ Clinic of Diagnostic Imaging, Department of Clinical Services, Vetsuisse Faculty University of Zurich Zurich Switzerland

**Keywords:** DGGR, dogs, gastrointestinal ultrasonography, lipase activity, MCAI, pancreatic ultrasonography, pancreatitis, UPASS

## Abstract

**Background:**

It is unclear if dogs with acute pancreatitis differ clinically from dogs with non‐pancreatic acute gastrointestinal disease (aGId).

**Objectives:**

Compare clinical findings in dogs with acute gastrointestinal signs suspected of having acute pancreatitis (sAP) based on increased DGGR‐lipase activity versus those with presumptive aGId.

**Animals:**

Twenty‐six dogs with sAP, 48 dogs with aGId based on acute signs, lipase activity > 450 U/L (RI, 17–156 U/L) and within/minimally (20 U/L) > RI, respectively.

**Methods:**

Prospective study. Clinical signs were graded using a simplified modified clinical activity index (MCAI). CBC, biochemistry, C‐reactive protein (CRP), pancreatic, and gastrointestinal ultrasonographic findings were compared between groups.

**Results:**

Median (range) disease duration before presentation (sAP 36 h [3–96 h], aGId 48 h [3–168 h]) did not differ. Diarrhea was significantly more frequent in aGId; MCAI did not differ between groups. Median (range) lipase activities in sAP and aGId dogs were 1280 U/L (451–6712) and 49.5 U/L (14–176), respectively. Alkaline phosphatase activity and bilirubin were significantly higher in sAP. Pancreatic ultrasonographic abnormalities were significantly more common in sAP. In aGId, a mixed‐echoic (17/44, 39%), hyperechoic (9/44, 20%), hypoechoic pancreas (3/44, 7%), and hyperechoic mesentery (4/44, 9%) were found. Only a distended stomach was significantly more common in sAP. Multivariable logistic regression analysis only identified pancreatic enlargement and ultrasonographic diagnosis of pancreatitis to increase the odds of sAP. Hospitalization (median, range) did not differ (sAP 3, 1–8 days; aGId 2.5, 1–5 days).

**Conclusion and Clinical Importance:**

Both groups do not differ in clinical severity; diarrhea is less prevalent, and mild cholestasis is more common in sAP. Pancreatic ultrasonographic changes suggestive of AP are rare in aGId.

**Abbreviations**
aGId: acute gastrointestinal disease
AP: acute pancreatitis
CRP: C‐reactive protein concentration
DGGR: 1,2‐o‐dilauryl‐rac‐glycero‐3‐glutaric acid‐(6 0‐methylresorufin) ester
MCAI: modified canine activity index
sAP: suspected acute pancreatitis
UPASS: ultrasonographic pancreatic assessment severity score

## 1 Introduction

Suspected acute pancreatitis (sAP) is frequent in dogs. The diagnostic gold standard histopathology carries a high risk of morbidity and potentially mortality; therefore, AP is diagnosed clinically [1]. Reported signs include vomiting, abdominal pain, anorexia, lethargy, and diarrhea [2, 3, 4, 5, 6, 7, 8], dogs might only present with one clinical sign [3]. Diarrhea varies in frequency from 25% to 63% [2, 3, 4, 5, 6, 7, 8], while diarrhea is not a clinical sign of AP in people [9].

Dogs with acute gastrointestinal disease (aGId, formerly acute gastroenteritis) present with similar signs [10], specifics have only been published for acute hemorrhagic diarrhea syndrome [11, 12]. Clinical signs of sAP and aGId have not been compared. Serum lipase is considered the most important laboratory marker for AP in dogs and measured as an activity (DDGR‐based catalytic lipase assays) [13] or concentration using an immunoassay (pancreatic lipase immunoreactivity [PLI]) [14]. DGGR‐lipase and PLI assays correlate strongly with each other [3, 13, 15, 16, 17, 18, 19, 20, 21]. Quasi‐identical results of the Roche (DGGR‐based) lipase activity [13] and PLI assay were described when lipase was measured daily in dogs with AP during hospitalization [3]. Magnitude and nature of change were the same for both assays [3], illustrating that the LIPC Roche assay is by no means less specific than the PLI assay. Routine laboratory findings of both conditions, including C‐reactive protein concentration (CRP) have also never been compared. CRP is increasingly reported in both, AP [3, 22] and aGId [11, 23]. It is unknown whether both diseases differ in terms of severity of inflammation.

Typical ultrasonographic AP features include a hypoechoic, enlarged pancreas with or without a hyperechoic mesentery [4, 24]. There is no consensus on how many abnormal ultrasonographic findings are needed for an imaging diagnosis of pancreatitis. Importantly, pancreatic lesions might become apparent only after 2–3 days in acute disease stages or can also remain absent [25, 26]. Therefore, an ultrasonographically normal pancreas in acutely sick dogs does not rule out early stages or milder forms of AP [24, 25, 26]. The same has been found in people [27]. Moreover, pancreatitis is more likely to be diagnosed ultrasonographically if dogs have had a previous pancreatitis episode [18, 22]. When such dogs present for acute non‐pancreatic gastrointestinal disease, residual ultrasonographic pancreatic changes might be interpreted as causative pancreatitis. Furthermore, it is unknown what the pancreas looks like in dogs with aGId.

Since lipase and ultrasonography results are not always immediately available, we wanted to know whether there are further differences between diseases. Therefore, we aimed to compare (i) clinical signs and severity, (ii) CBC, serum biochemistry, and CRP results, and (iii) pancreatic and selected gastrointestinal tract ultrasonographic parameters between AP and aGId. Our hypotheses were: (a) dogs with aGId present more frequently with diarrhea, (b) clinical severity between diseases does not differ, and (c) ultrasonographic changes suspicious for AP are rarely found in aGId.

## 2 Material and Methods

### 2.1 Study Cohort

Client‐owned dogs presented between 04/2023 and 03/2024 to the Clinic for Small Animal Internal Medicine, Vetsuisse Faculty, University of Zurich with sAP or aGId were considered for study inclusion. Inclusion criteria for sAP were an increased DGGR‐lipase activity [13] > 450 U/L (LIPC, Roche on Cobas c501, Roche Diagnostics; RI, 17–156 U/L) together with an acute onset (< 7 days) of ≥ 2 of the following clinical signs suspicious for AP: vomiting, abdominal pain, lethargy, anorexia, and diarrhea, and no other explanation for hyperlipasemia. Dogs with a normal or minimally increased (< 20 U/L) lipase activity and acute onset (< 7 days) of the aforementioned clinical signs without apparent causes for clinical signs were allocated to the aGId group. Our current LIPC Roche lipase activity RI (17 U/L [90% CI 12–21]–156 [90% CI 129–224] U/L) is higher than the previous RI (24–108 U/L) [13]. This new RI is based on 123 clinically healthy dogs aged between 1 and 12 years, fasted for 6–8 h (measured on Cobas c501, Roche) [28]. Exclusion criteria were major concurrent diseases such as intestinal obstruction, abdominal mass, cardiopulmonary disease, liver failure, metabolic disease, autoimmune disease, severe anemia, systemic infection, neoplastic disease, acute kidney injury, or chronic kidney disease (CKD) IRIS stage > 2.

The study was approved by the Cantonal Veterinary office of Zurich and conducted in accordance with guidelines established by the Animal Welfare Act of Switzerland (No ZH029/2023).

### 2.2 Study Design

Prospective cross‐sectional study. Standardized medical histories were taken in all dogs. Owners were specifically questioned regarding disease duration before presentation in hours and known previous pancreatitis episodes. When dogs presented with concurrent vomiting and diarrhea, owners were specifically asked what occurred first. In the hospital, the presence of appetite, vomiting, diarrhea, abdominal pain, dehydration, lethargy, and blood in feces was assessed and graded using a slightly simplified canine activity index (MCAI). Minor modifications were pursued in terms of appetite, abdominal pain, and fecal consistency (appetite graded as normal [=0], decreased [=1], absent [=2]; abdominal pain graded as none [=0], suspicion of abdominal pain [=1], abdominal pain [=2]; fecal consistency graded as normal [=0], soft or poorly formed [=1], or watery diarrhea [=2]). Hematologic variables (Sysmex X N‐1000) involved hematocrit, platelets, segmented neutrophils, and lymphocytes. Blood smear microscopy assessed neutrophil toxicity and the presence of band neutrophils. Serum biochemistry variables (Cobas c501, Roche) comprised glucose, total protein, albumin, alkaline phosphatase activity (ALP), alanine transaminase activity, bilirubin, creatinine, urea, triglycerides, cholesterol, sodium, potassium, chloride, calcium, and phosphate. CRP was measured using the Gentian Canine CRP Immunoassay (RI, 0–10.2 mg/dL).

The following pancreatic ultrasonographic (LOGIQ E10, GE Healthcare) variables were assessed: pancreatic enlargement, echogenicity (hyperechoic, hypoechoic), echotexture (homogeneous, mixed‐echoic), mesenteric echogenicity, and free peripancreatic fluid. To assess the ultrasonographic severity of pancreatitis a modified ultrasonographic pancreatic assessment severity score (UPASS) [29] was calculated. A minor modification was that a normal mesentery was scored with 0, whereas a hyperechogenic mesentery was scored with 1. UPASS ranged from 0 (no changes) to maximally 6. Presence of an ultrasonographic diagnosis of pancreatitis based on the final diagnosis in the radiology report containing either “pancreatitis,” “suspicion of pancreatitis,” or “pancreatopathy” regardless of severity or chronicity was recorded. Evaluated gastrointestinal ultrasonographic variables were presence of a distended stomach, corrugated duodenum, aperistaltic intestines, peritoneal effusion, and mesenteric lymphadenopathy. All but one ultrasonographic examination were performed by board‐certified diagnostic imagers or a resident‐in‐training under direct supervision of a board‐certified diagnostic imager. One examination was performed by a board‐certified internist experienced in abdominal ultrasonography.

### 2.3 Statistical Analysis

The software SAS 9.4 (SAS Institute Inc.) was used for statistical analysis. Normality of parametrical datasets was confirmed by QQ‐Plots. Analysis comprised fixed effect models (group, sex, age, weight) of laboratory variables that met the precondition of normality, applying the procedure GLM. In the case of bilirubin and glucose, which did not meet the precondition of normality as raw nor transformed data, generalized linear models (group, sex, interactions) assuming a log‐link function over a gamma distribution were mapped. Age and weight were not considered in these analyses since the inclusion of these variables did not improve the model fit. Frequency of clinicopathological variables between groups was assessed by Fisher's exact and Chi‐square (*χ* ^2^) tests (procedure FREQ). Spearman's rank correlation coefficients (*r*_*s*_) were used to assess correlations between the UPASS or an ultrasonographic diagnosis of pancreatitis and the age of the sAP and aGId dogs or previous pancreatitis episodes. Correlation coefficients were determined for the following: MCAI and CRP, and UPASS, and also CRP and UPASS, as well as the duration of disease in hours and lipase activity, and CRP. Mann–Whitney *U* tests were used to compare MCAI, lipase activity, CRP, and UPASS between dogs with and without individual clinical signs and with and without individual ultrasonographic findings.

To address the concern regarding multiple comparisons and to assess the multifactorial impact of clinical, laboratory, and ultrasonographic variables, a multivariable logistic regression approach was performed with the procedure LOGISTIC. First, each of 69 potential predictors (clinical signs, laboratory, ultrasonographic findings) was screened in a univariate logistic regression model to assess its association with the binary outcome of dogs being associated with either group sAP or aGId. Variables with *p* ≤ 0.10 in univariate testing were retained as candidates for multivariable regression. Multivariable logistic regression was fitted by backward stepwise selection, applying again a cutoff of *p* ≤ 0.10 to remain in the final model. This two‐step approach ensured that only variables showing at least nominal evidence of association were considered further, thereby limiting risks of overfitting. Adjusted odds ratios (OR) and 95% confidence intervals (CI) were estimated for each variable retained in the final multivariable model. Model fit was assessed using the Hosmer–Lemeshow Goodness‐of‐Fit Test.

## 3 Results

### 3.1 Dogs

Twenty‐six dogs were included in the sAP and 48 dogs in the aGId group. Signalments are shown in Table 1. Dogs with sAP were significantly older, with a median age of 7.4 (range, 0.4–14.4) compared to 4.9 years (range, 0.6–11.8) in aGId (*p* = 0.02). Three sAP (12%) and 7 (15%) aGId dogs had a history of a previous pancreatitis episode. Six dogs were excluded because lipase values were neither within sAP nor aGId cut‐offs.

**TABLE 1:** Signalment of sAP (acute clinical signs suspicious of AP and DGGR lipase activity > 450 U/L) aGId dogs (same clinical signs and DGGR lipase activity within RI or minimally increased [< 20 U/L]).

		sAP dogs (*n* = 26)	aGId dogs (*n* = 48)	*p*
Sex	*n* (%)	10 female (38)	20 female (42)	0.81
		16 male (62)	28 male (58)	
Age (years)	Median (range)	7.4 (0.4–14.4)	4.9 (0.6–11.8)	0.02^a^
Weight (kg)	Median (range)	8.7 (1.2–38.0)	10.5 (2.0–49.7)	0.39
Breed	(*n*)	Mixed breed (5), Chihuahua (4), German Spitz (3), Poodle (3), Jack Russel Terrier (2), Australian Cobberdog (1), Bernese Mountain Dog (1), Bolonka (1), Cocker Spaniel (1), Dachshund (1), Flat Coated Retriever (1), Greyhound (1), Labrador Retriever (1), Welsh Terrier (1)	Dachshund (7), Mixed breed (5), German Spitz (3), Labrador Retriever (3), Miniature Schnauzer (3), Poodle (3), Havanese (2), Magyar Viszla (2), Australian Shephard (1), Barsoi (1), Basset Hound (1), Beagle (1), Bolonka (1), Border Terrier (1), Entlebuch Mountain Dog (1), Flat Coated Retriever (1), German Shephard (1), Goldendoodle (1), Lagotto Romagnolo (1), Maltese (1), Podenco Canario (1), Prague Rattler (1), Pug (1), Rhodesian Ridgeback (1), Serbian Hound (1), Weimaraner (1), Welsh Corgi Pembroke (1), Yorkshire Terrier (1)	

*Note:* This table denotes Mann Whitney *U*‐test comparisons of clinicopathological variables (sex, age, and weight). An alpha level of 0.05 was used to determine statistical significance.

^a^ Statistically significant value.

### 3.2 Presenting Clinical Signs

Presenting clinical signs are summarized in Table 2. SAP dogs presented with vomiting (21/26, 81%), anorexia (19/26, 73%), lethargy (15/26, 58%), and diarrhea (15/26, 58%). AGId dogs presented with vomiting (44/48, 92%), diarrhea (40/48, 83%), and anorexia (31/48, 65%). Diarrhea was significantly more common the presenting sign in aGId compared to sAP (*p* = 0.03).

**TABLE 2:** Comparison of presenting clinical signs between sAP (acute clinical signs suspicious of AP and DGGR lipase activity > 450 U/L) and aGId dogs (same clinical signs and DGGR lipase activity within RI or minimally increased [< 20 U/L]).

Presenting clinical signs	sAP dogs (*n* = 26)	aGId dogs (*n* = 48)	*p*
Vomiting	21/26 (81%)	44/48 (92%)	0.26
Of which hematemesis	4/21 (19%)	8/44 (18%)	1.0
Diarrhea	15/26 (58%)	40/48 (83%)	0.03^a^
Of which bloody diarrhea	11/15 (73%)	24/40 (60%)	0.77
Lethargy	15/26 (58%)	28/48 (58%)	1.0
Anorexia	19/26 (73%)	31/48 (65%)	0.6
Abdominal pain	7/26 (27%)	6/48 (13%)	0.2
Restlessness	8/26 (31%)	7/48 (15%)	0.13
Increased body temperature	1/26 (4%)	0/48 (0%)	0.35
Tenesmus	3/26 (12%)	3/48 (6%)	0.66
Weight loss	1/26 (4%)	4/48 (8%)	0.65
Coughing	1/26 (4%)	0/48 (0%)	0.35
Hypersalivation	0/26 (0%)	2/48 (4%)	0.54
Borborygmus	0/26 (0%)	1/48 (2%)	1.0
Hematochezia	1/26 (4%)	2/48 (4%)	1.0
PU/PD	1/26 (4%)	0/48 (0%)	0.35

*Note:* This table denotes Fisher's exact test comparisons of clinicopathological variables. An alpha level of 0.05 was used to determine statistical significance.

^a^ Statistically significant value.

Twelve (46%) sAP and 37 (77%) aGId dogs presented with both vomiting and diarrhea. In one dog per group, owners could not determine whether vomiting or diarrhea began first. In the remaining 11 and 36 dogs with concomitant vomiting and diarrhea, diarrhea was more often the first clinical sign in aGId (22/36, 61%) compared to sAP (4/11, 36%). This difference did not reach statistical significance (*p* = 0.18).

Frequencies of bloody admixtures in vomit within vomiting (sAP 4/21 dogs, 19%; aGId 8/44 dogs, 18%) and in diarrhea within diarrheic dogs (sAP 11/15, 73%; aGId 24/40, 60%) did not significantly differ between groups. Only one sAP dog presenting without diarrhea developed diarrhea later during hospitalization.

### 3.3 Associations of Presenting Clinical Signs With MCAI, Lipase Activity, CRP, and UPASS

**FIGURE 1:**
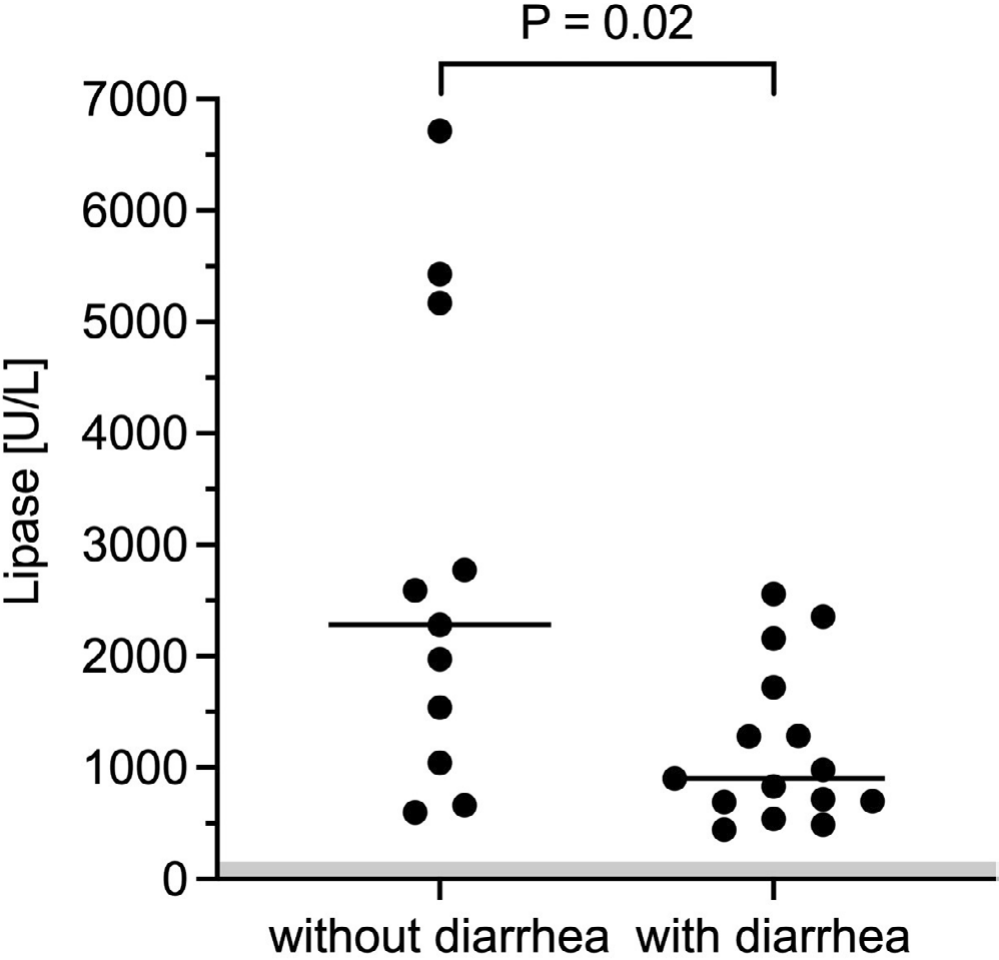
sAP dogs (acute clinical signs suspicious of AP and lipase activity > 450 U/L) without diarrhea (*n* = 11) had significantly higher lipase activities compared to dogs with diarrhea (*n* = 15). An alpha level of 0.05 was used to determine statistical significance.

**FIGURE 2:**
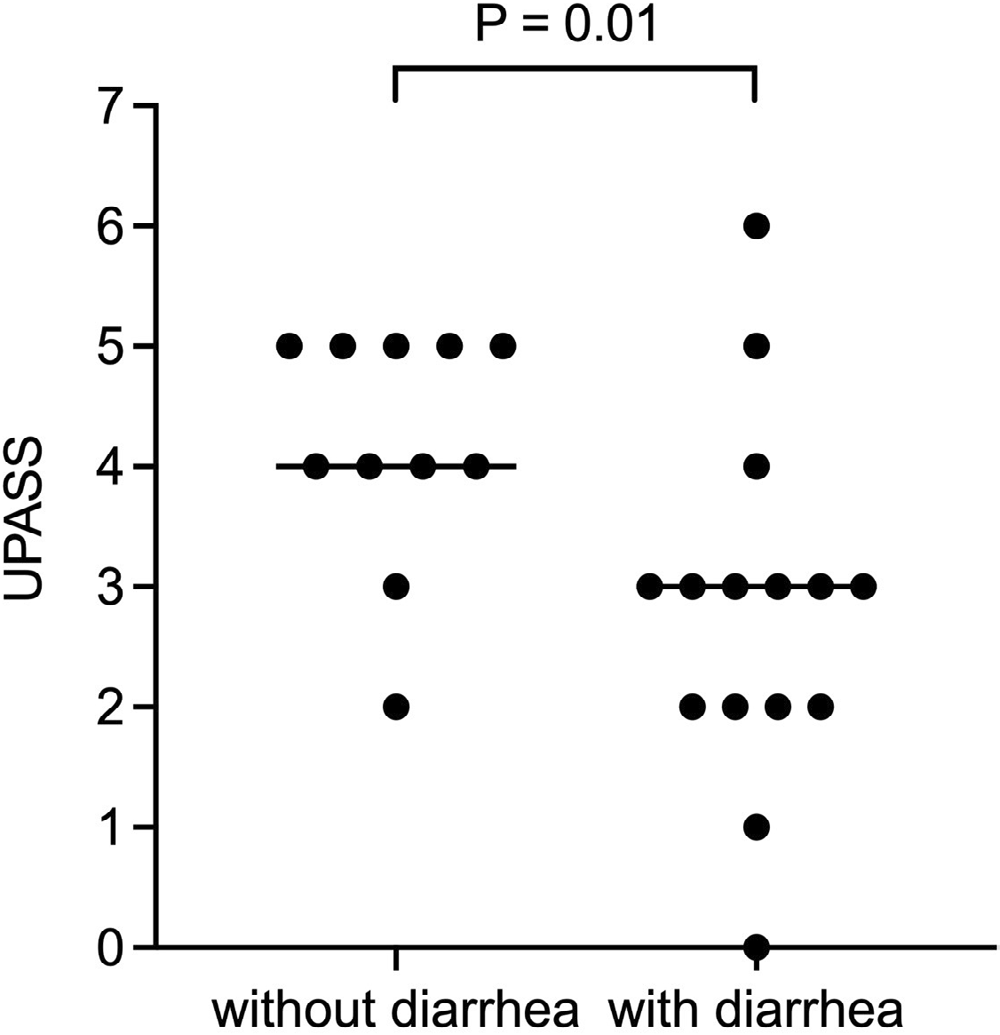
The ultrasonographic pancreatic assessment severity score (UPASS) was significantly higher in sAP dogs (acute clinical signs suspicious of AP and lipase activity > 450 U/L) without diarrhea compared to AP dogs with diarrhea. An alpha level of 0.05 was used to determine statistical significance.

SAP dogs without diarrhea (*n* = 11) had significantly higher lipase activities (median, 2279 U/L; range, 599–6712) compared to dogs with diarrhea (*n* = 15; median, 902 U/L; range, 451–2558; *p* = 0.02; Figure 1). Similarly, UPASS was significantly higher in sAP dogs without diarrhea (median = 4, range, 2–5) compared to diarrheic sAP dogs (median = 3, range, 0–6; *p* = 0.01; Figure 2). SAP dogs presenting with vomiting (*n* = 21) had significantly increased CRP (median, 69.2 mg/L; range, 6.4–370.0) compared to sAP dogs without vomiting (*n* = 5; median, 9.0 mg/L, range 6.4–86.8; *p* = 0.03). Dogs with sAP and restlessness (*n* = 8) had significantly increased lipase activities (median, 2666 U/L; range, 661–6712) compared to sAP dogs without restlessness (*n* = 18; median, 1012 U/L; range, 451–2591; *p* = 0.02). Lipase activity, CRP, total MCAI, and UPASS were not significantly different when abdominal pain, lethargy, or anorexia were present or absent.

Median duration of clinical signs before presentation was 36 h (range, 3–96) in sAP and 48 h (range, 3–168) in aGId dogs, respectively, and not significantly different. Of the sAP dogs, 12 presented within 24, 8 within 48, 5 within 72, and 1 within 96 h. Of the aGId dogs, 17 presented within 24, 14 within 48, 10 within 96, and 7 after 96 h. Lipase activity did not correlate with disease duration before presentation in both groups.

### 3.4 Clinical Severity of Disease

The individual MCAI components are shown in Table 3 and did not differ between groups. Total MCAI, representing the overall clinical severity, was also not significantly different with a median of 5 (range, 0–10) in sAP and 6 (range, 0–13) in aGId.

**TABLE 3:** Comparison of the single scores of the modified canine activity index (MCAI) at baseline between sAP (acute clinical signs suspicious of AP and DGGR lipase activity > 450 U/L) and aGId dogs (same clinical signs and DGGR lipase activity within RI or minimally increased [< 20 U/L]).

Single scores		sAP dogs (*n* = 26)	aGId dogs (*n* = 48)	*p*
General demeanor	0	4/26 (15%)	10/48 (21%)	0.26
	1	13/26 (50%)	30/48 (63%)	
	2	9/26 (35%)	8/48 (17%)	
	3	0/26 (0%)	0/48 (0%)	
Appetite	0	13/26 (50%)	13/48 (27%)	0.15
	1	6/26 (23%)	19/48 (40%)	
	2	7/26 (27%)	16/48 (33%)	
Vomiting	0	22/26 (85%)	36/48 (75%)	0.56
	1	1/26 (4%)	7/48 (15%)	
	2	1/26 (4%)	2/48 (4%)	
	3	2/26 (8%)	3/48 (6%)	
Abdominal pain	0	6/26 (23%)	18/48 (38%)	0.47
	1	9/26 (35%)	15/48 (31%)	
	2	11/26 (42%)	15/48 (31%)	
Dehydration	0	20/26 (77%)	28/48 (58%)	0.13
	1	0/26 (0%)	0/48 (0%)	
	2	6/26 (23%)	20/48 (42%)	
	3	0/26 (0%)	0/48 (0%)	
Fecal consistency	0	6/18 (33%)	4/35 (11%)	0.07
	1	1/18 (6%)	1/35 (3%)	
	2	11/18 (61%)	30/35 (86%)	
	No DEF	8/26	13/48	
Blood in feces	0	10/18 (56%)	16/35 (46%)	0.5
	1	8/18 (44%)	19/35 (54%)	
	No DEF	8/26	13/48	

*Note:* This table denotes Fisher's exact test comparisons of clinicopathological variables. An alpha level of 0.05 was used to determine statistical significance.

Abbreviation: DEF, defecation.

Median duration of hospitalization in sAP (3 days; range, 1–8) and aGId dogs (2.5 days; range, 1–5) was not significantly different. In the sAP group, 25/26 (96%) dogs survived and were discharged, while all aGId dogs survived. The 73 dogs that survived hospital discharge were alive 7 days after said discharge.

### 3.5 Laboratory Findings

**FIGURE 3:**
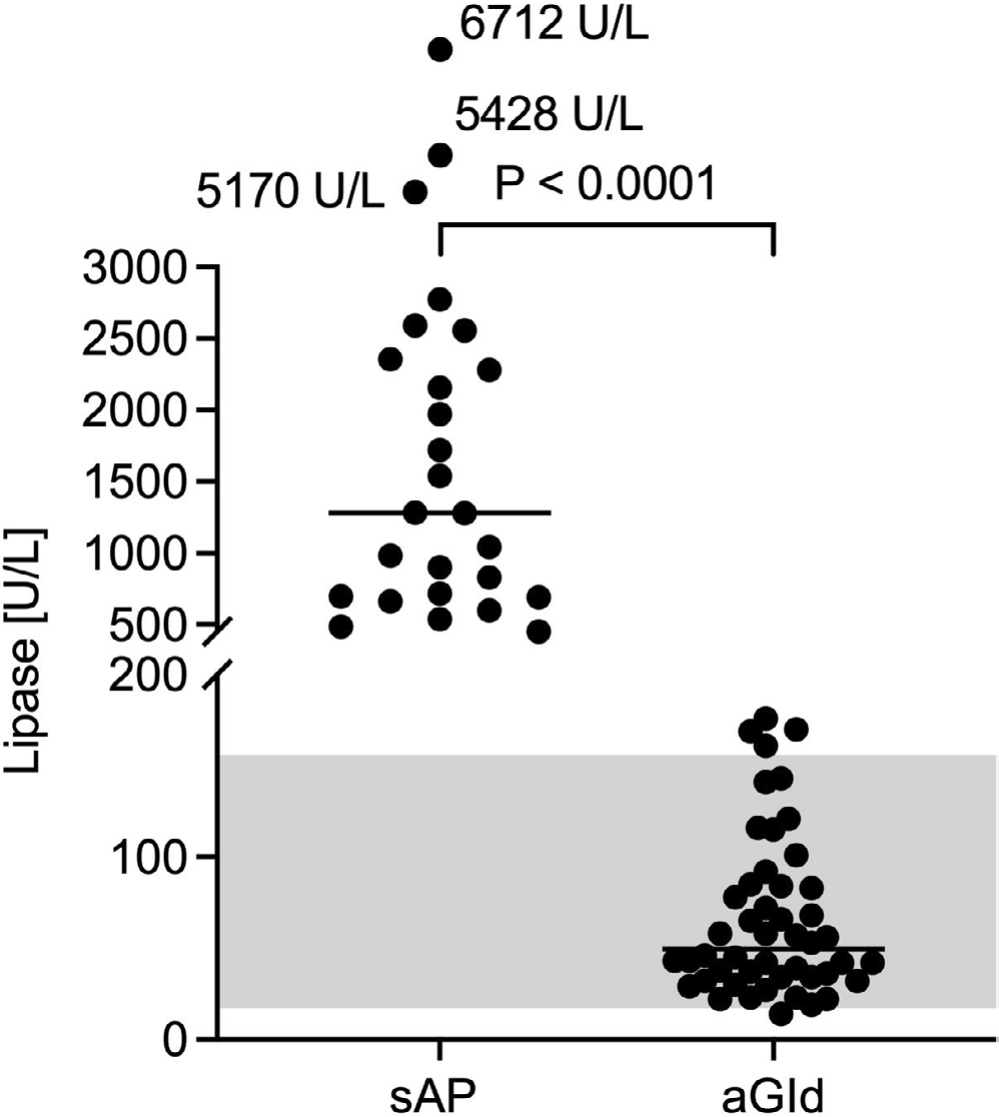
Median lipase activities were 1280 U/L (range, 451–6712 U/L) in sAP (acute clinical signs suspicious of AP and lipase activity > 450 U/L) and 49.5 U/L (range 14–176 U/L) in aGId (same clinical signs and lipase activity within RI or minimally increased [< 20 U/L]). An alpha level of 0.05 was used to determine statistical significance.

**FIGURE 4:**
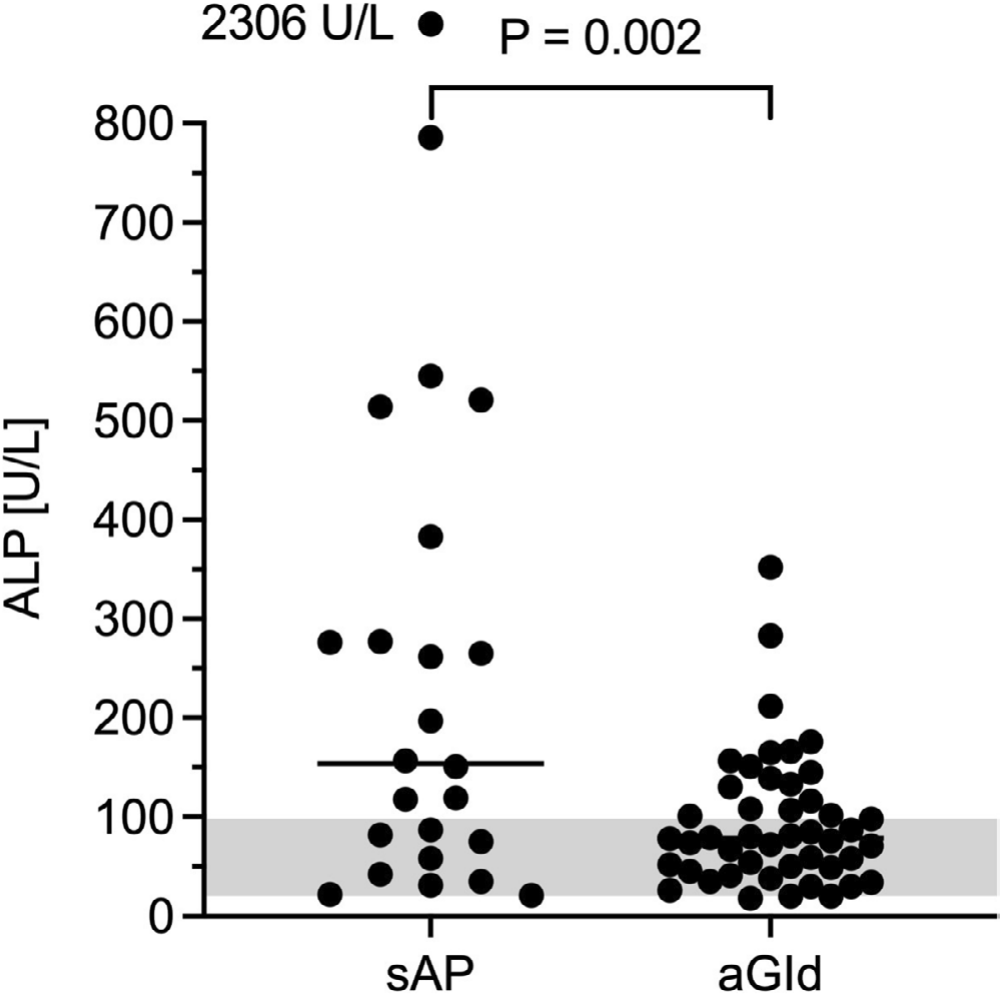
Alkaline phosphatase (ALP) activity was significantly higher in sAP (acute clinical signs suspicious of AP and lipase activity > 450 U/L) compared to aGId (same clinical signs and lipase activity within RI or minimally increased [< 20 U/L]). An alpha level of 0.05 was used to determine statistical significance.

**FIGURE 5:**
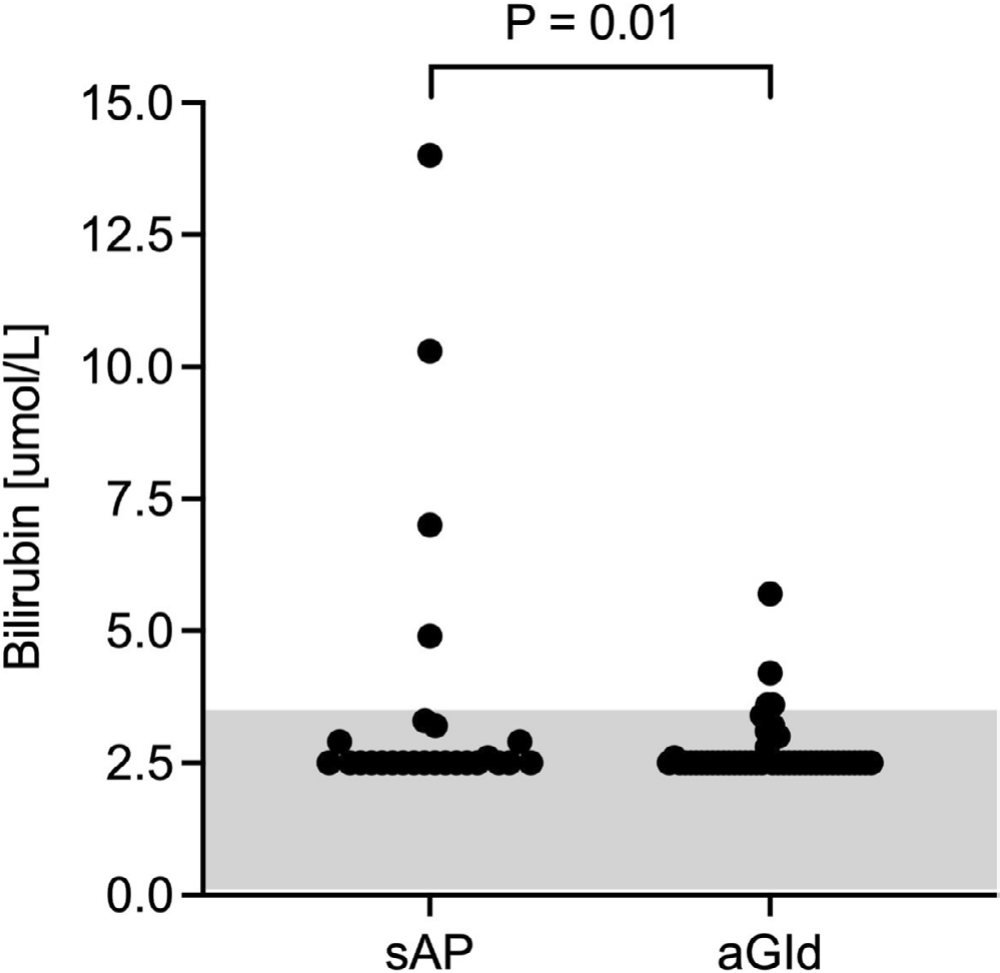
Bilirubin concentration was also significantly higher in sAP (acute clinical signs suspicious of AP and lipase activity > 450 U/L) compared to aGId same clinical signs and lipase activity within RI or minimally increased [< 20 U/L]). An alpha level of 0.05 was used to determine statistical significance.

**FIGURE 6:**
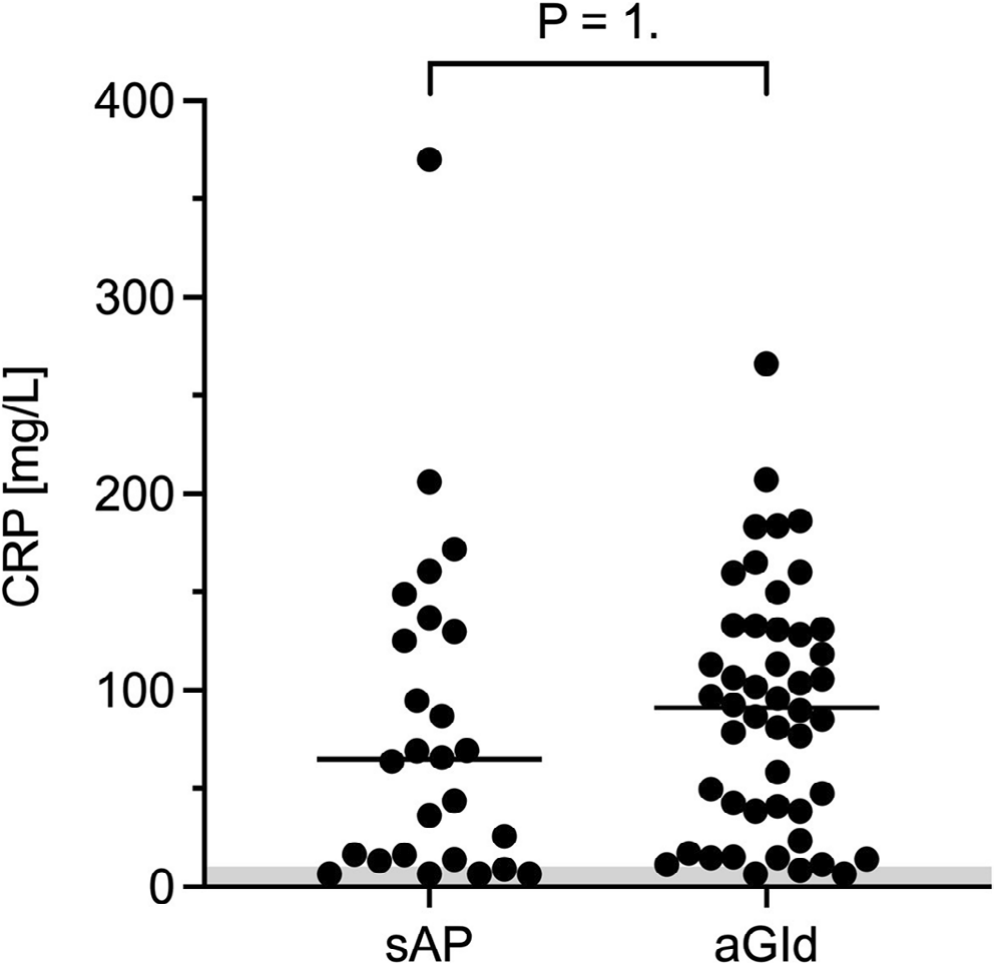
C‐reactive protein concentration (CRP) did not significantly differ between sAP (acute clinical signs suspicious of AP and lipase activity > 450 U/L) and aGId (same clinical signs and lipase activity within RI or minimally increased [< 20 U/L]). An alpha level of 0.05 was used to determine statistical significance.

CBC, biochemistry and CRP results are summarized in Tables 4 and 5. There were no significant differences in CBC parameters, including neutrophil toxicity assessment, between groups. Median lipase activities were 1280 U/L (range, 451–6712) in sAP and 49.5 U/L (range, 14–176) in aGId (Figure 3), respectively. ALP activity was significantly higher in sAP (median, 154 U/L, range, 21–2306; RI 20–98) compared to aGId (median, 78.5 U/L, range, 18–352; *p* = 0.002; Figure 4). Increased ALP activities were found in 63% (sAP) and 37% (aGId) of dogs. Bilirubin concentration was significantly higher in sAP (sAP median, 2.5 μmol/L, range, 2.5–14; aGId median, 2.5 μmol/L, range, 2.5–5.7; RI < 3.5 μmol/L; *p* = 01; Figure 5). Prevalence of hyperbilirubinemia was 15% and 9% in sAP and aGId, respectively. CRP did not significantly differ, with a median of 65 mg/L (range, 6.4–370.0; RI −10.2) in sAP and 91 mg/L (range, 6.4–266) in aGId dogs (Figure 6). Statistical significance was not reached for biochemical variables when the Bonferroni correction (adjusted significance level *p* = 0.0031) was applied.

**TABLE 4:** Comparison of hematologic parameters between sAP (acute clinical signs suspicious of AP and DGGR lipase activity > 450 U/L) and aGId dogs (same clinical signs and DGGR lipase activity within RI or minimally increased [< 20 U/L]) at presentation.

Analyte^a^		sAP dogs (*n* = 26)	aGId dogs (*n* = 48)	RI	*p*
Hematocrit	*n* dogs tested	24	45	42%–55%	0.22
	Median (range)	45% (33–60)	47% (37–59)		
	< 42%; *n* (%)	7 (29)	7 (16)		
	> 55%; *n* (%)	2 (8)	5 (11)		
Thrombocytes	*n* dogs tested	24	45	130–394 × 10^3^/μL	0.92
	Median (range)	297 × 10^3^/μL (117–580)	237 × 10^3^/μL (112–551)		
	< 130 × 10^3^/μL; *n* (%)	2 (8)	2 (4)		
	> 394 × 10^3^/μL; *n* (%)	3 (13)	2 (4)		
Segmented neutrophils	*n* dogs tested	25	47	2.5–7.44 × 10^3^/μL	0.21
	Median (range)	8.78 × 10^3^/μL (3.42–20.2)	7.67 × 10^3^/μL (1.5–27.43)		
	< 2.5 × 10^3^/μL; *n* (%)	0 (0)	4 (9)		
	> 7.44 × 10^3^/μL; *n* (%)	17 (68)	27 (57)		
Band neutrophils	*n* dogs tested	25	47	0–0.08 × 10^3^/μL	0.98
	Median (range)	0.26 × 10^3^/μL (0–4.98)	0.35 × 10^3^/μL (0–4.09)		
	> 0.08 × 10^3^/μL; *n* (%)	14 (56)	31 (66)		
Toxic neutrophils	*n* dogs tested	25	47	0	0.46
	Median (range)	0 (0–2)	0 (0–3)		
	> 0; *n* (%)	7 (28)	16 (34)		
Lymphocytes	*n* dogs tested	25	47	1.15–3.4 × 10^3^/μL	0.33
	Median (range)	1.23 × 10^3^/μL (0.13–6.33)	1.59 × 10^3^/μL (0.35–3.4)		
	< 1.15 × 10^3^/μL; *n* (%)	12 (48)	17 (36)		
	> 3.4 × 10^3^/μL; *n* (%)	2 (8)	0 (0)		

*Note:* This table denotes comparisons of clinicopathological variables using fixed effect models (group, sex, age, weight). An alpha level of 0.05 was used to determine statistical significance. Toxic neutrophils: 0 = 0%; 1 = 5%–10%; 2 = 11%–30%; 3 = > 30%.

Abbreviation: RI, reference interval.

^a^ For each parameter, the number (%) of cases above and below reference interval are also shown.

**TABLE 5:** Comparison of serum biochemistry including CRP between sAP (acute clinical signs suspicious of AP and DGGR lipase activity > 450 U/L) and aGId dogs (same clinical signs and DGGR lipase activity within RI or minimally increased [< 20 U/L]) at presentation.

Analyte^a^		sAP dogs (*n* = 26)	aGId dogs (*n* = 48)	RI	*p*
Lipase	*n* dogs tested	26	48	17–156 U/L	< 0.0001^b^
	Median (range)	1280 U/L (451–6712)	49.5 U/L (14–176)		
	< 17 U/L; *n* (%)	0 (0)	1 (2)		
	> 156 U/L; *n* (%)	26 (100)	4 (8)		
CRP	*n* dogs tested	26	48	−10.2 mg/L	1.0
	Median (range)	64.85 mg/L (6.4–370)	91.15 mg/L (6.4–266.2)		
	> 10.2 mg/L; *n* (%)	21 (81)	45 (94)		
Glucose	*n* dogs tested	25	46	4.1–5.9 mmol/L	0.33
	Median (range)	5.9 mmol/L (4.4–25.2)	6.1 mmol/L (4.1–8.9)		
	< 4.1 mmol/L; *n* (%)	0 (0)	0 (0)		
	> 5.9 mmol/L; *n* (%)	12 (48)	30 (65)		
Total protein	*n* dogs tested	25	46	56–71 g/L	0.18
	Median (range)	57 g/L (33–69)	49.5 g/L (29–81)		
	< 56 g/L; *n* (%)	10 (40)	33 (72)		
	> 71 g/L; *n* (%)	0 (0)	1 (2)		
Albumin	*n* dogs tested	25	46	29–37 g/L	0.25
	Median (range)	34 g/L (21–45)	30 g/L (17–47)		
	< 29 g/L; *n* (%)	5 (20)	17 (37)		
	> 37 g/L; *n* (%)	8 (32)	6 (13)		
ALP	*n* of dogs tested	24	46	20–98 g/L	0.002^b^
	Median (range)	154 U/L (21–2306)	78.5 U/L (18–352)		
	< 20 g/L; *n* (%)	0 (0)	1 (2)		
	> 98 g/L; *n* (%)	15 (63)	17 (37)		
ALT	*n* dogs tested	25	46	20–93 U/L	0.23
	Median (range)	56 U/L (16–496)	35 U/L (19–357)		
	< 20 g/L; *n* (%)	1 (4)	2 (4)		
	> 93 g/L; *n* (%)	5 (20)	2 (4)		
Bilirubin	*n* dogs tested	26	46	< 2.5–3.5 μmol/L	0.01^b^
	Median (range)	2.5 μmol/L (2.5–14)	2.5 μmol/L (2.5–5.7)		
	> 3.5 μmol/L; *n* (%)	4 (15)	4 (9)		
Creatinine	*n* dogs tested	25	46	50–119 μmol/L	0.16
	Median (range)	63 μmol/L (17–118)	54 μmol/L (22–135)		
	< 50 μmol/L; *n* (%)	6 (24)	20 (43)		
	> 119 μmol/L; *n* (%)	0 (0)	1 (2)		
Urea	*n* dogs tested	25	46	3.8–9.4 mmol/L	0.86
	Median (range)	5.1 mmol/L (2.5–13)	4.3 mmol/L (1.9–21)		
	< 3.8 mmol/L; *n* (%)	5 (20)	17 (37)		
	> 9.4 mmol/L; *n* (%)	3 (12)	2 (4)		
Triglycerides	*n* dogs tested	25	46	0.4–1.5 mmol/L	0.39
	Median (range)	0.6 mmol/L (0.3–14.9)	0.55 (0.3–1.1)		
	< 0.4 mmol/L; *n* (%)	3 (12)	3 (7)		
	> 1.5 mmol/L; *n* (%)	3 (12)	0 (0)		
Cholesterol	*n* dogs tested	25	46	3.5–8.6 mmol/L	0.28
	Median (range)	4.8 mmol/L (3.1–12.5)	4.1 mmol/L (2.1–10.3)		
	< 3.5 mmol/L; *n* (%)	4 (16)	11 (24)		
	> 8.6 mmol/L; *n* (%)	2 (8)	2 (4)		
Sodium	*n* dogs tested	25	46	145–152 mmol/L	0.95
	Median (range)	146 mmol/L (129–153)	145.5 mmol/L (136–153)		
	< 145 mmol/L; *n* (%)	5 (20)	13 (28)		
	> 152 mmol/L; *n* (%)	2 (8)	1 (2)		
Potassium	*n* dogs tested	24	46 4.2 mmol/L (3.2–4.9)	4.3–5.3 mmol/L	0.39
	Median (range)	4.2 mmol/L (3.5–4.9)			
	< 4.3 mmol/L; *n* (%)	14 (58)	24 (52)		
	> 5.3 mmol/L; *n* (%)	0 (0)	0 (0)		
Total calcium	*n* of dogs tested	25	46	2.4–2.8 mmol/L	0.07
	Median (range)	2.51 mmol/L (1.95–2.97)	2.28 mmol/L (1.86–3.08)		
	< 2.4 mmol/L; *n* (%)	9 (36)	30 (65)		
	> 2.8 mmol/L; *n* (%)	1 (4)	1 (2)		
Phosphate	*n* of dogs tested	24	46	1–1.6 mmol/L	0.15
	Median (range)	1.25 mmol/L (0.75–2.46)	1.15 mmol/L (0.73–2.5)		
	< 1 mmol/L; *n* (%)	2 (8)	12 (26)		
	> 1.6 mmol/L; *n* (%)	6 (25)	4 (9)		

*Note:* This table denotes comparisons of clinicopathological variables using fixed effects models (group, sex, age, weight). Glucose and Bilirubin did not meet the precondition of normality and were analyzed by generalized mixed models assuming a log‐link function over a gamma distribution (group, sex). An alpha level of 0.05 was used to determine statistical significance. When the Bonferroni correction was applied, an adjusted significance level of 0.0031 was calculated for the total of 16 comparisons of the serum biochemistry parameters, invalidating the statistical significance of all findings. CRP: For statistical analysis, values < 6.4 mg/L were depicted as 6.4 mg/L. Bilirubin: For statistical analysis, values < 2.5 μmol/L were depicted as 2.5 μmol/L.

Abbreviations: ALP, alkaline phosphatase; ALT, alanine aminotransferase; CRP, C‐reactive protein; RI, reference interval.

^a^ For each parameter, the number (%) of cases above and below the reference interval is also shown.

^b^ Statistically significant value.

### 3.6 Ultrasonographic Findings

Ultrasonography was performed in all sAP dogs and in 44/48 (92%) aGId dogs. Results including UPASS are shown in Tables 6 and 7. Median time between lipase measurement and ultrasound was 6 h (range, 0–54) in the sAP and 3 h (range, 0–51) in the aGId group, respectively. Median (range) UPASS was 3 in sAP (0–6) and 0 (0–3) in aGId. All pancreatic ultrasonographic variables were significantly more common in sAP. All but one sAP dog (96%) had a final ultrasonographic diagnosis of pancreatitis. The one dog without an ultrasonographic diagnosis of pancreatitis had a mixed‐echoic pancreas and thus an UPASS of 1.

**TABLE 6:** Comparison of pancreatic ultrasonographic variables between sAP (acute clinical signs suspicious of AP and DGGR lipase activity > 450 U/L) and aGId dogs (same clinical signs and DGGR lipase activity within RI or minimally increased [< 20 U/L]).

Pancreatic ultrasonographic variables		sAP dogs (*n* = 26)	aGId dogs (*n* = 44)	*p*
Pancreatic enlargement	Absent	12/26 (46%)	44/44 (100%)	< 0.0001^a^
	Present	14/26 (54%)	0/44 (0%)	
Pancreatic echogenicity	Normal	4/26 (15%)	32/44 (73%)	< 0.0001^a^
	Hyperechoic	6/26 (23%)	9/44 (20%)	
	Hypoechoic	16/26 (62%)	3/44 (7%)	
Pancreatic echotexture	Homogeneous	6/26 (23%)	28/44 (64%)	0.004^a^
	Mixed‐echoic	20/26 (77%)	17/44 (39%)	
Hyperechoic mesentery	Absent	12/26 (46%)	40/44 (91%)	< 0.0001^a^
	Present	14/26 (54%)	4/44 (9%)	
Peripancreatic free fluid	Absent	23/26 (89%)	44/44 (100%)	0.048^a^
	Present	3/26 (12%)	0/44 (0%)	
Total UPASS	0	0/26 (0%)	23/44 (52%)	< 0.0001^a^
	1	2/26 (8%)	11/44 (25%)	
	2	5/26 (19%)	5/44 (11%)	
	3	7/26 (27%)	5/44 (11%)	
	4	5/26 (19%)	0/44 (0%)	
	5	6/26 (23%)	0/44 (0%)	
	6	1/26 (4%)	0/44 (0%)	
Pancreatic ultrasonographic diagnosis	Absent	1/26 (4%)	33/44 (75%)	< 0.0001^a^
	Present	25/26 (96%)	11/44 (25%)	

*Note:* This table denotes Fisher's exact test comparisons of diagnostic imaging variables. An alpha level of 0.05 was used to determine statistical significance.

^a^ Statistically significant value.

**TABLE 7:** Comparison of selected gastrointestinal ultrasonographic findings between sAP (acute clinical signs suspicious of AP and DGGR lipase activity > 450 U/L) and aGId dogs (same clinical signs and DGGR lipase activity within RI or minimally increased [< 20 U/L]).

GI ultrasonographic findings		sAP dogs (*n* = 26)	aGId dogs (*n* = 44)	*p*
Distended stomach	Absent	1/26 (4%)	10/44 (23%)	0.16
	Mildly	14/26 (54%)	16/44 (36%)	
	Moderately	8/26 (31%)	14/44 (32%)	
	Severely	3/26 (12%)	4/44 (9%)	
Corrugated duodenum	Absent	23/26 (88%)	40/44 (91%)	1.0
	Present	3/26 (12%)	4/44 (9%)	
Aperistaltic intestines	Absent	21/26 (81%)	33/44 (75%)	0.77
	Present	5/26 (19%)	11/44 (25%)	
Peritoneal effusion	Absent	16/26 (62%)	34/44 (77%)	0.08
	Present	10/26 (39%)	10/44 (23%)	
Mesenteric lymphadenopathy	Absent	13/25 (52%)	21/44 (48%)	0.8
	Present	12/25 (48%)	23/44 (52%)	
	LN not visualized	1/26		

*Note:* This table denotes Fisher's exact test comparisons of diagnostic imaging variables. An alpha level of 0.05 was used to determine statistical significance.

Abbreviation: LN, lymph nodes.

Within the sAP group, lipase activity was significantly higher when an enlarged pancreas was present with a median of 2256 U/L (range, 451–6712) in dogs with pancreatic enlargement (*n* = 14) and a median of 707.5 U/L (range, 486–2279) in dogs without an enlarged pancreas (*n* = 12; *p* = 0.0008). CRP and total MCAI were not significantly different between sAP dogs with or without pancreatic enlargement. Lipase activity, CRP, and total MCAI did not differ significantly when a hypoechoic or hyperechoic pancreas, a hyperechoic mesentery, or peripancreatic free fluid were present or absent.

In aGId dogs, a mixed‐echoic (17/44, 39%), hyperechoic (9/44, 20%), and hypoechoic (3/44, 7%) pancreas was found. In addition, 4/44 (9%) dogs had a hyperechoic mesentery. Pancreatic enlargement was not observed. Eleven of 44 (25%) aGId dogs had an ultrasonographic diagnosis of pancreatitis. Four of these 11 dogs (36%) had a known previous pancreatitis episode. Disease duration, presenting signs, lipase activities, presence of pancreatic ultrasonographic variables, and UPASS of 11 aGId dogs with an ultrasonographic diagnosis of pancreatitis are shown in Table S1. In all 11 dogs (100%), lipase activity remained within RI during the entire hospitalization period (data not shown).

UPASS and an ultrasonographic diagnosis of pancreatitis correlated significantly with age in aGId (UPASS and age, *r*_*s*_ = 0.549, *p* = 0.0001; ultrasonographic diagnosis of pancreatitis and age, *r*_*s*_ = 0.362, *p* = 0.02) but not in sAP dogs. AGId dogs with an ultrasonographic diagnosis of pancreatitis were significantly older, with a median of 6.8 years (range, 2.6–11.8) compared to 4.5 years (range, 0.6–9.8) in aGId dogs without US diagnosis of pancreatitis (*p* = 0.02). Furthermore, UPASS correlated significantly with a known previous pancreatitis episode in aGId dogs (*r*_*s*_ = 0.364, *p* = 0.02). Given the low prevalence of previous pancreatitis episodes, this was not calculated in the sAP group.

Frequencies of a distended stomach, corrugated duodenum, aperistaltic intestines, peritoneal effusion, and mesenteric lymphadenopathy were not different between groups (Table 7). If a broader classification (present versus absent) was used for the variable “distended stomach” instead of mildly to severely filled, a distended stomach was significantly more prevalent in sAP (AP 96%; aGId 77%; *p* = 0.04).

### 3.7 Correlations Between Lipase Activity, CRP, UPASS, and Clinical Severity

Correlations between lipase activity, CRP, UPASS, and clinical severity are summarized in Table 8. CRP and UPASS correlated positively in sAP (*r*_*s*_ = 0.416, *p* = 0.03) but not in aGId. CRP and total MCAI did not correlate significantly in both groups. Total MCAI and UPASS did not correlate significantly in both groups. Neither lipase activity nor CRP correlated significantly with the duration of hospitalization in both groups.

**TABLE 8:** Spearman's rank correlation coefficient (*r*_*s*_) and statistical significance (*p* value) for the correlations between lipase activity, CRP, ultrasonographic pancreatic assessment severity score (UPASS), and modified canine activity index (MCAI) in sAP (acute clinical signs suspicious of AP and DGGR lipase activity > 450 U/L) and aGId dogs (same clinical signs and DGGR lipase activity within RI or minimally increased [< 20 U/L]).

Group	MCAI and CRP	MCAI and UPASS	Lipase and duration of hospitalization	CRP and duration of hospitalization	CRP and UPASS
sAP	*r*_*s*_ = −0.005, *p* = 0.98	*r*_*s*_ = −0.107, *p* = 0.6	*r*_*s*_ = 0.269, *p* = 0.19	*r*_*s*_ = 0.066, *p* = 0.75	*r*_*s*_ = 0.416, *p* = 0.03^a^
aGId	*r*_*s*_ = 0.176, *p* = 0.23	*r*_*s*_ = 0.144, *p* = 0.35	*r*_*s*_ = −0.184, *p* = 0.21	*r*_*s*_ = 0.187, *p* = 0.2	*r*_*s*_ = −0.124, *p* = 0.42

*Note:* An alpha level of 0.05 was used to determine statistical significance.

^a^ Statistically significant value.

### 3.8 Predictors of sAP in Dogs

A multivariable logistic regression was performed to identify predictors for dogs being diagnosed with sAP. The final model included pancreatic enlargement (absent vs. present) and ultrasonographic diagnosis of pancreatitis (absent vs. present). The intercept was statistically significant (estimate = −3.43, *p* = 0.0007). Pancreatic enlargement was significantly associated with a higher odds of sAP (OR = 12.0; 95% CI [1.3, 110.53]; *p* = 0.03; Table 9). A final ultrasonographic diagnosis of pancreatitis was also significantly associated with sAP (OR = 31.0; 95% CI [3.52, 273.04]; *p* = 0.03; Table 9).

**TABLE 9:** Multivariable logistic regression of clinicopathological variables regarding the odds of dogs being associated with the group sAP with a cutoff of *p* ≤ 0.10 after backward selection.

Variable	DF	Estimation	Standard error	Wald's chi‐square	Pr > Chisq	OR (95% CI)
Intercept	1	−3.43	1.02	11.42	0.0007^a^	
Pancreatic enlargement (present vs. absent)	1	2.48	1.13	4.81	0.03^a^	12. (1.3–110.53)
US diagnosis of pancreatitis (present vs. absent)	1	4.43	1.11	9.57	0.002^a^	31. (3.52–273.04)

*Note:* Multivariable logistic regression included pre‐screening of 69 clinical, laboratory, and ultrasonographic variables tested in 75 dogs for their association with group sAP versus aGId via univariate logistic regression with a cutoff of *p* ≤ 0.10. The identified variables were used in a subsequent multivariable logistic regression with backward selection, applying again a cutoff of *p* ≤ 0.10. An alpha level of 0.05 was used to determine statistical significance.

Abbreviations: CI, confidence interval; DF, degrees of freedom; OR, odds ratio; US, ultrasonographic.

^a^ Statistically significant value.

## 4 Discussion

The present study compares clinical, laboratory, and ultrasonographic findings of dogs with sAP and aGId. We deliberately divided both groups according to lipase levels because we also wanted to compare pancreatic ultrasonographic findings between groups. Besides, it is well documented that lipase levels (lipase activities and PLI) and pancreatic ultrasound findings do not agree well [3, 13, 18, 22, 29, 30]. Dogs without lipase increases can indeed have ultrasonographic pancreatic changes [13, 18] while dogs in the very acute disease stage might not yet have any changes [25, 26]. In addition, previous pancreatitis episodes are associated with ultrasonographic evidence of pancreatopathy [18, 22] and it is reasonable to assume that especially milder acute pancreatitis episodes are often not diagnosed as such. Moreover, specificity of ultrasonographic findings commonly attributed to “acute pancreatitis” has not been investigated before. We have chosen a lipase activity cutoff approximately > 3× the upper RI limit, a standard concept used to diagnose AP in humans and dogs [31, 32]. A similar approach was followed when the “consistent with pancreatitis” threshold of the PLI assay RI (400 μL) was implemented.

In our study, the only clinical sign differing between groups was diarrhea. Diarrhea was significantly more prevalent in aGId; however, it was also present in 58% of sAP dogs. This is in contrast to AP in people, where abdominal pain is the primary clinical sign and diarrhea is not a sign [9]. A possible explanation for diarrhea in the sAP group might be a secondary or concurrent pancreatitis in dogs primarily affected by acute enteritis. Gastrointestinal fluid losses might lead to poor tissue perfusion, resulting in pancreatic ischemia, which could trigger the development of AP. It is well documented in humans that the pancreas is vulnerable to ischemic injury, and pancreatic hypoperfusion due to various causes can lead to AP [33, 34]. Increased lipase activity and PLI results have been reported in puppies and young dogs with acute enteritis and parvoviral enteritis [35, 36]. Another explanation could be that diarrhea is indeed a clinical sign of AP in dogs. Duodenal microbiome perturbations occur secondary to AP in people [37] and intestinal dysbiosis might be involved in the disease progression [38, 39]. Experimentally disrupted pancreatic function with subsequently altered pancreatic juice secretion resulted in intestinal dysbiosis in mice [40]. Maybe changes in the duodenal microbiome secondary to AP serve as an etiological factor for the occurrence of diarrhea in dogs.

We were interested in whether lipase activity, CRP, total MCAI, and UPASS differed in dogs with sAP when individual clinical signs were present or absent. SAP dogs presenting without diarrhea had significantly higher lipase activities and UPASS. A recent study showed that amongst all reported clinical signs, only the presence of lethargy, vomiting, and abdominal pain was significantly associated with lipase activity in dogs presented for gastrointestinal clinical signs [18]. It was suggested that these signs best characterize the clinical presentation of AP in dogs [18]. In light of this finding, it could be assumed that dogs with suspicion of pancreatitis without diarrhea are more likely to be suffering from a primary AP. This would explain the higher lipase activities observed in these patients. This interpretation is supported by the fact that only one sAP dog presenting without diarrhea developed diarrhea later on during hospitalization. As stated above, sAP dogs presenting with diarrhea had a significantly lower UPASS. Possibly, it takes more time in secondary AP until ultrasonographic pancreatic changes appear compared to primary AP, or pancreatic damage is so mild that it cannot be visualized ultrasonographically.

Clinical severity as assessed by the total MCAI and duration of hospitalization did not differ between groups. All dogs with aGId survived to discharge; one (4%) sAP dog died suddenly and unexpectedly on the 3rd day of hospitalization.

When comparing laboratory values, ALP activity and bilirubin concentration were significantly higher in sAP. These findings suggest that mild cholestasis is more common in AP, which could be explained by the close anatomical proximity of the inflamed pancreas and the biliary system. Extrahepatic cholestasis secondary to an acute and acute‐on‐chronic episode of pancreatitis is well‐known [4, 41] and usually indicates more severe disease [41]. Laboratory findings seen in aGId have only been described in dogs with acute hemorrhagic diarrhea syndrome [12], but pancreatic enzymes were not measured in that study. Compared to our findings in aGId dogs, a similar prevalence of hyperbilirubinemia but lower prevalence of increased ALP activity was reported [12].

Inflammatory markers, including band and toxic neutrophils as well as CRP, did not significantly differ between sAP and aGId. Thus, the severity of the inflammatory response of both diseases appears to be comparable; also, CRP did not discriminate sAP from aGId. CRP did not correlate with clinical severity at presentation in both groups, indicating that the usefulness of CRP determination as a biomarker for disease severity is limited. Moreover, CRP did not correlate with the duration of hospitalization in both groups. Our results indicate that CRP levels at hospital admission have limited prognostic value, which has previously been shown in dogs with AP [42, 43, 44] and aGId [11, 23].

Our findings illustrate that ultrasonographic findings of the pancreas encountered in aGId dogs reflect mostly chronic changes, with hyper‐ (20%) and/or mixed‐echoic (39%) echogenicities being the most common. Comparable information, such as 7% hyperechoic and 40% mixed‐echoic pancreatic echogenicities, has been reported in healthy dogs [45]. Similarly, the only other study that examined the pancreas in dogs without gastrointestinal signs found nonhomogeneous pancreatic echotexture and hyperechoic foci in 8% of dogs [46]. Those dogs had no clinical suspicion of pancreatitis and underwent ultrasonographic evaluation for various reasons [46]. They had a median age of 12–14 years, which led authors to conclude that chronic pancreatic ultrasonographic changes are age‐related [46]. This agrees with our findings, as both the UPASS and the ultrasonographic diagnosis of pancreatitis correlated positively with age in aGId dogs. In humans without pancreatic disease, pancreatic echogenicity also increases with age [47]. Hyperechoic pancreata were detected ultrasonographically in 35% of people < 40 years of age; this prevalence increased to 65% when > 61 years old [47]. The fact that a bit more than a third of aGId dogs with an ultrasonographic diagnosis of pancreatitis also had a known previous pancreatitis episode might also suggest the possibility of concurrent chronic pancreatitis. However, this is difficult to prove in very acutely sick dogs that improve quickly with supportive treatment.

It is currently unknown how often adult dogs with acute intestinal disease have pancreatic ultrasonographic changes. We are reporting for the first time prevalences of a hypoechoic (9%) pancreas. Ultrasonographic changes consistent with AP have only been reported in puppies with parvovirus enteritis [35]. Dogs in that study were classified based on PLI > 400 or < 400 μg/L into two groups [35]. Acute pancreatic ultrasonographic changes, not described in detail, were reported in 1/18 (6%) dogs with PLI < 400 μg/L, which would correspond to our aGId group [35].

A hyperechoic mesentery was detected ultrasonographically in 11% of aGId dogs, which is also a novel finding. So far, ultrasonographic evidence of steatitis in dogs with aGId has been reported in a dog affected by acute enterocolitis without suspicion of AP [48] and in dogs with intussusception due to acute gastroenteritis [49]. A hyperechoic mesentery has also been reported in cats with acute intestinal disease [50, 51] and is commonly detected in humans with acute appendicitis [52]. Our findings suggest that ultrasonographic evidence of peritoneal inflammation can also be present in dogs with acute enteritis, although concurrent mild pancreatitis cannot be completely ruled out as a cause. This aligns with the clinical experiences of our senior radiologists and has also been mentioned sporadically in radiology textbooks for dogs with gastrointestinal disease [53], but not yet reported in clinical studies.

We were also interested in whether lipase activity, CRP, and total MCAI differ between sAP dogs with or without respective pancreatic ultrasonographic changes. A significantly higher lipase activity was only found in sAP dogs with pancreatic enlargement compared to those without. No aGId dog had an enlarged pancreas, suggesting a high specificity of this ultrasonographic finding. Moreover, multivariable logistic regression analysis revealed that the presence of an enlarged pancreas is a strong predictor for dogs being diagnosed with sAP. This is similar to a previous study in which pancreatic enlargement showed most associations with clinical signs and had the highest effect size of all US findings that were significantly associated with lipase activity in dogs [13]. Pancreatic size was also significantly associated with UPASS in another study [29]. Since the pancreas is not routinely measured like the adrenal glands, for example, there is still uncertainty as to when the pancreas is actually considered enlarged. In addition, the size of dogs can also play a role here. We therefore assume that ultrasonographic pancreatic changes are already at a more advanced stage when diagnostic imagers consider the pancreas to be enlarged. Still, the presence of an enlarged pancreas seems a significant ultrasonographic predictor of canine AP.

So far, no studies provide information on the ultrasonographic appearance of the gastrointestinal tract in dogs with AP and aGId. Of all selected gastrointestinal ultrasonographic variables, only a distended stomach was significantly more common in sAP when categorized into present versus absent. Nearly all sAP dogs (96%) displayed a distended stomach, reflecting some form of gastric stasis. Gastric wall thickening, presumably because of edema, has recently been reported in dogs with AP and was discussed to be the consequence of direct extension of the inflammatory process to the gastric submucosa [54]. The close proximity of extra‐gastric and pancreatic lymphatic vessels was discussed as a cause [54]. However, 75% of aGId dogs also had some form of gastric distension, and the provided inflammation theory might apply to both diseases, especially as there were no differences in the laboratory markers of inflammation between both groups. Future studies are needed to specifically examine gastric emptying times in dogs with AP and aGId.

Our study had some limitations. The unequal group sizes might have affected results. It was difficult to recruit equal numbers for both groups because the present data were collected as part of another project where blood samples had to be frozen to −80°C immediately. This made inclusion of sAP cases more difficult, as they tended to come in more often on weekends. Another reason for fewer sAP dogs was that the presence of relevant concurrent disease was an exclusion criterion. As aGId dogs were younger, this was less of a problem in this group. By using fixed‐effect models and GLMs, we were able to control for potential confounding variables such as sex, age, and weight, and to handle data that did not meet normality assumptions. These statistical methods are robust to differences in group sizes and variances, allowing for valid comparisons despite unequal sample sizes. Additionally, we employed non‐parametric tests to analyze variables that did not meet parametric assumptions. These methods are less sensitive to sample size discrepancies and allowed us to make valid comparisons across groups. While we recognize that residual errors might persist and that no single study can comprehensively address all questions, we believe that by transparently sharing our findings, our data can be incorporated into future meta‐analyses. This would enable more powerful analyses across multiple studies, ultimately contributing to informed decisions and recommendations for clinical practice.

Secondly, we cannot rule out the possibility that some aGId dogs also had mild inflammatory processes in the pancreas, as the final differentiation of groups was based on lipase measurements and no test is 100% accurate.

Thirdly, abdominal ultrasounds were performed by different radiologists, which likely resulted in varying interpretations. This certainly applies to subjective graduation criteria such as mild or moderate gastric filling but also to pancreatic variables. Ultimately, it is unknown when and based on how many abnormal ultrasonographic variables an ultrasonographic diagnosis of pancreatitis is made by radiologists. This is why we also used the UPASS in order to be less dependent on ultrasonographic diagnoses of pancreatopathy/pancreatitis. Ideally, all dogs would have been examined by the same radiologist, but this was not possible in our workflow. However, we believe our approach is a realistic representation of everyday clinical practice in a referral center. Furthermore, we were able to include cases very early in the disease course compared to available studies reporting specifics on disease duration [3, 7, 55]. This is why we were apprehensive of potentially missing numerous AP cases if we had used both inclusion criteria, increased lipase activity and a minimum of two ultrasonographic features of AP. Moreover, we acknowledge that pretreatments might have affected some of our results; however, it is very difficult to control these factors in a clinical setting.

Lastly, we cannot exclude without histopathology the possibility that some dogs might have had concurrent chronic pancreatitis and there might have been some overlap between groups. Some aGId dogs might have also had mild pancreatic inflammation, both of which would require a histopathological examination for confirmation. Besides ultrasonographic results, this might have also affected lipase results and the frequency of clinical signs. However, all dogs were still acutely sick at the time of inclusion. In this context, it should also be mentioned that it is very difficult to accurately record previous pancreatitis episodes. It is possible that owners have simply forgotten or acute episodes of gastrointestinal disease have not been diagnosed as AP by private veterinarians.

In conclusion, we could not detect clinical differences between sAP and aGId except that sAP dogs present less frequently with diarrhea. Also, the clinical severity of disease does not differ between sAP and aGId. With the exception of mild cholestasis being more common in sAP, routine laboratory findings do not differ between groups. This also applies to all inflammatory markers assessed. Pancreatic ultrasonographic changes in dogs with aGId likely reflect chronic changes and are similar in frequency to what is seen in healthy dogs [47]. Ultrasonographic pancreatic changes suggestive of AP are rare in dogs with minimal lipase activity increase. An enlarged pancreas and ultrasonographic diagnosis of pancreatitis significantly increase the odds of suspected AP. Future studies are warranted to examine whether our results are reproducible when aGId dogs with an ultrasonographically completely normal pancreas are compared to sAP dogs with pre‐defined ultrasonographic pancreatic abnormalities.

## Disclosure

Authors declare no off‐label use of antimicrobials.

## Ethics Statement

Institutional animal care and use committee approved by the cantonal veterinary office of Zurich, Switzerland. Authors declare human ethics approval was not needed.

## Conflicts of Interest

The authors declare no conflicts of interest.

## Supporting information


**Table S1.** Duration of disease, laboratory, and imaging findings including ultrasonographic pancreatic assessment severity score (UPASS) in aGId dogs with an ultrasonographic diagnosis of pancreatitis.
