# Supplementary material for: Comparison of Clinical, Laboratory, and Ultrasonographic Findings in Dogs With Acutely Presenting Clinical Signs and Either Normal or Increased Serum DGGR Lipase Activity
Source: J Vet Intern Med. 2025 Jun 1;39(4):e70134. doi: 10.1111/jvim.70134 (PMC12127568; doi:10.1111/jvim.70134)
Supplement: Supplementary file 1 — Table S1. Duration of disease, laboratory, and imaging findings including ultrasonographic pancreatic assessment severity score (UPASS) in aGId dogs with an ultrasonographic diagnosis of pancreatitis. [file JVIM-39-e70134-s001.docx]

**Table S1.** Duration of disease, laboratory, and imaging findings including ultrasonographic pancreatic assessment severity score (UPASS) in aGId dogs with an ultrasonographic diagnosis of pancreatitis.

| Dog | Duration of disease [h] | Presenting clinical signs | Lipase [U/L] | Pancreatic ultrasonographic variables | UPASS |
| --- | --- | --- | --- | --- | --- |
| 1 | 48 | Vomiting, bloody diarrhea, hypo-/anorexia | 58 | Hyperechoic, mixed-echoic pancreas + hyperechoic mesentery | 3 |
| 2 | 24 | Vomiting, bloody diarrhea, lethargy, hypo-/anorexia | 32 | Hyperechoic, mixed-echoic pancreas | 2 |
| 3 | 36 | Vomiting, bloody diarrhea, lethargy, hypo-/anorexia | 66 | Hyperechoic, mixed-echoic pancreas + hyperechoic mesentery | 3 |
| 4 | 48 | Vomiting, diarrhea, hypo-/anorexia, abdominal pain | 29 | Hypoechoic pancreas + hyperechoic mesentery | 3 |
| 5 | 24 | Vomiting, lethargy, abdominal pain, restlessness | 83 | Hypoechoic pancreas | 2 |
| 6 | 12 | Hematemesis, bloody diarrhea | 43 | Hyperechoic pancreas | 1 |
| 7 | 12 | Vomiting, restlessness, tenesmus, hematochezia | 72 | Mixed-echoic pancreas | 1 |
| 8 | 12 | Vomiting, diarrhea, lethargy | 161 | Hyperechoic, mixed-echoic pancreas + hyperechoic mesentery | 3 |
| 9 | 72 | Vomiting, bloody diarrhea, hypo-/anorexia | 32 | Mixed-echoic pancreas | 1 |
| 10 | 9 | Vomiting, lethargy | 167 | Hyperechoic pancreas | 2 |
| 11 | 96 | Vomiting, bloody diarrhea, lethargy, hypo-/anorexia, restlessness | 68 | Hyperechoic, mixed-echoic pancreas | 2 |
